# The chemopreventive potential of lycopene against atrazine-induced cardiotoxicity: modulation of ionic homeostasis

**DOI:** 10.1038/srep24855

**Published:** 2016-04-26

**Authors:** Jia Lin, Hui-Xin Li, Jun Xia, Xue-Nan Li, Xiu-Qing Jiang, Shi-Yong Zhu, Jing Ge, Jin-Long Li

**Affiliations:** 1 College of Veterinary Medicine, Northeast Agricultural University, Harbin, 150030, People’s Republic of China; 2 Division of Avian Infectious Diseases, State Key Laboratory of Veterinary Biotechnology, Harbin Veterinary Research Institute, Chinese Academy of Agricultural Sciences, Harbin, People’s Republic of China

## Abstract

People who drink water contaminated with atrazine (ATR) over many years can experience problems with their cardiovascular system. Lycopene (LYC) has been shown to exhibit cardiovascular disease preventive effects. However, chemopreventive potential of LYC against ATR-induced cardiotoxicity remains unclear. To determine the effects of ATR and/or LYC on heart, mice were treated with ATR (50 mg/kg or 200 mg/kg) and/or LYC (5 mg/kg) by intragastric administration for 21 days. Histopathological and biochemical analyses, including analysis of ion concentrations (Na^+^, K^+^, Ca^2+^ and Mg^2+^), ATPases (Na^+^-K^+^-ATPase, Ca^2+^-ATPase, Mg^2+^-ATPase and Ca^2+^-Mg^2+^-ATPase) activities and the transcription of their subunits, were performed on heart. The results revealed that ATR led to decreased Creative Kinase (CK) activity and increased histological alterations. Furthermore, a significant change in Na^+^, K^+^ and Ca^2+^ content and the down-regulation of Na^+^-K^+^-ATPase and Ca^2+^-ATPase activities and the mRNA expression of their subunits were observed in ATR-exposed mice. Notably, supplementary LYC significantly protected the heart against ATR-induced damage. In conclusion, ATR induced cardiotoxicity by modulating cardiac ATPase activity and the transcription of its subunits, thereby triggering ionic disturbances. However, supplementary LYC significantly combated ATR-induced cardiotoxicity via the regulation of ATPase activity and subunit transcription. Thus, LYC exhibited a significant chemopreventive potential against ATR-induced cardiotoxicity.

Atrazine (ATR) is an herbicide used extensively to control broadleaf and grassy weeds on crops such as corn, sorghum, and sugarcane. With approximately 73–78 million pounds being applied per year, ATR is one of the most commonly applied pesticides in the United States. As a result of its high mobility and persistence in water, ATR is frequently detected in streams, rivers and groundwater in many countries. ATR concentrations in surface waters could reach up to 300 μg L^−1^ in Iowa and some other places. Currently, the adverse effects of this herbicide on human and animal health are not fully understood.

Cardiovascular diseases (CVD) are the leading causes of human morbidity and mortality. Exposure to atrazine (ATR) can lead to severe cardiac damage, which may result in CVD^1^,^2^,^3^,^4^. ATR has been one of the most widely used herbicides in recent decades. Due to its widespread presence and continued use in most countries, there are increasing concerns about the potential adverse health effects of ATR. ATR and/or its metabolites (such as atrazine-desethyl-desisopropyl; DACT) have been detected in heart tissues^5^, and ATR contribute to cardiovascular disorders during intoxication^1^,^2^.

Previous studies have shown that the effects of ATR are mostly caused by oxidative stress^6^,^7^. During the last decade, these adverse effects induced by ATR, including oxidative stress and endocrine disruption, have been extensively studied. Despite great efforts in studying the toxicity of ATR to the heart, relatively little consideration have been given to the toxicity of ionic disorders. Indeed, abnormal concentrations of K^+^, Na^+^ and Ca^2+^, indicating a water-electrolyte imbalance, may result in cardiac arrhythmias and muscle contraction disorders^8^.

Lycopene (LYC), a naturally occurring hydrocarbon carotenoid that is found in red foods such as tomatoes, pink guavas, watermelons, and papayas, has attracted considerable attention as a potential chemopreventive agent against disorders such as CVD in humans^9^. The numerous conjugated double bonds of LYC makes this compound a powerful antioxidant. Indeed, LYC has been shown to be a very efficient singlet oxygen quencher *in vitro*, as well as a scavenger of other reactive oxygen species (ROS) such as superoxide radicals, peroxyl radicals, and hydroxyl radicals^10^. Thus, previous studies have proposed that LYC may be important in preventing CVD through a mechanism related to antioxidative effects. There is also increasing evidence to suggest that LYC improves blood flow and reduces inflammatory responses^11^. Indeed, LYC regulates markers of inflammation, lipid status, thrombosis and oxidative stress to establish CVD preventive effects^12^, and these mechanisms may explain the cardio-protective effects of tomatoes and tomato products^13^. Considerable evidence has suggested that differences in ion levels may be partially responsible for arrhythmias or the incidence of death resulting from cardiovascular events^14^,^15^. Thus, maintenance of ionic balance is essential for cardiac function. Nevertheless, little is known about the role of LYC in the regulation of ionic homeostasis and the underlying chemopreventive mechanisms of LYC during ATR exposure.

Proper ionic balance plays a critical role in cardiac function^8^, and ATR has been demonstrated to cause cardiac toxicity. However, the relationship between cardiac ionic disorder and ATR-induced cardiotoxicity remains unclear. LYC is known to decrease the risk of certain chronic diseases, especially CVD and cancer^15^,^16^, and the potential protective role of LYC on the heart has been described in previous studies^11^,^12^,^17^. However, little is known about the protective effects of LYC on cardiac function through the maintenance of ionic equilibrium and consequent reduction of cardiac toxicity during ATR exposure. Therefore, the aim of this study was to investigate the association between ATR-induced cardiotoxicity and the chemopreventive potential of LYC on the heart and to assess whether modulation of the cardiac ionic balance is involved in these effects. Finally, we offer a conclusion on the chemopreventive potential of LYC in heart health and the amelioration of ATR-induced cardiac toxicity.

## Results

### Body and cardiac weights

There were no significant alterations in body weight or the relative weight of the heart in the ATR and/or LYC treatment groups compared to the control group after 21 days of treatment (see Supplementary Table S1). The animals in all groups remained in relatively good health throughout the study, and there were no exposure-related clinical observations. These results demonstrated few changes in body weight in the ATR and/or LYC treatment groups.

### Histopathological and biochemical analyses of heart tissues

**Figure 1:**
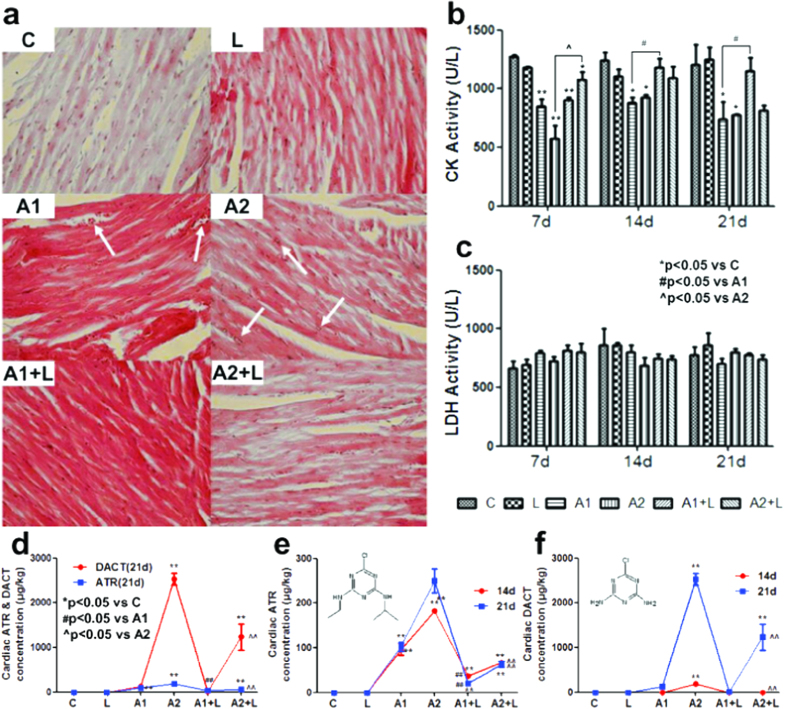
Effects of ATR and/or LYC on the histopathological, biochemical analysis and ATR & DACT concentration in mouse heart. (**a**) The histopathological analysis in heart, the arrows in white point to the location of the lesion; (**b**) The CK activity in serum; (**c**) The LDH activity in serum; (**d**) The ATR & DACT concentration in heart at 21 days; (**e**) The ATR concentration in heart at 14 & 21 days; (**f**) The DACT concentration in heart at 14 & 21 days. Values were expressed as mean ± S.D. Symbol for the significance of differences between the vehicle control and another: *P < 0.05, **P < 0.01. Symbol for the significance of differences between the 50 mg/kg ATR-treated group and 50 mg/kg ATR +5 mg/kg LYC treatment group: ^#^*P* < 0.05, ^##^*P* < 0.01. Symbol for the significance of differences between the 200 mg/kg ATR-treated group and 200 mg/kg ATR +5 mg/kg LYC treatment group: ^^^*P* < 0.05, ^^^^*P* < 0.01.

Histological analysis of heart sections obtained from control animals revealed normal cytoarchitecture of the organ after 21 days of treatment. However, significant hyperaemia was observed in 50 mg/kg ATR-exposed mice, while significant vesicular degeneration occurred in 200 mg/kg ATR-exposed mice at 21 days (Fig. 1a). In addition, all indicators in the supplemental LYC group were normal compared to the control group (Fig. 1a). These results indicated that the effects of ATR on cardiac histological damage could be ameliorated by the administration of LYC + ATR.

The effects of ATR and/or LYC administration on the cardiac damage index were evaluated according to the expression of marker enzymes such as lactate dehydrogenase (LDH) and creatinine kinase (CK) in serum samples from control and treated mice. Biochemical changes in the serum after ATR and/or LYC treatment at 7 days, 14 days and 21 days are shown in Fig. 1b,c. The activity of LDH was not markedly different among the groups. ATR exposure significantly decreased the CK activity at 7 days, 14 days and 21 days (*P* < 0.05) compared with the control group. In contrast, the CK activity was significantly elevated in the LYC + ATR (200 mg/kg) group compared to the ATR (200 mg/kg) group (*P* < 0.05) at 7 days. Additionally, the CK activity was significantly increased in the LYC + ATR (50 mg/kg) group compared to the ATR (50 mg/kg) group (*P* < 0.05) at 14 days and 21 days. Thus, according to the cardiac CK activity, the administration of LYC + ATR showed a beneficial effect.

### ATR and DACT concentration in heart tissues

There were significantly increased levels of ATR and DACT in the hearts of mice exposed to ATR compared to animals in the control group (Fig. 1d–f). In comparison to the control group, the ATR and DACT levels were increased in animals exposed to ATR but were reduced in those that were pre-treated with LYC. Moreover, it appeared that DACT and ATR were partially cleared from the heart as a result of LYC treatment for 21 days. The ATR and DACT levels were higher in animals that were exposed to ATR for 21 days than those exposed for 14 days (Fig. 1e,f). The maximum DACT and ATR levels were found to be dose-dependent and time-dependent in the heart (Fig. 1).

### Detection of K^+^ content, Na^+^-K^+^-ATPase activity and ATPase subunit transcription

**Figure 2:**
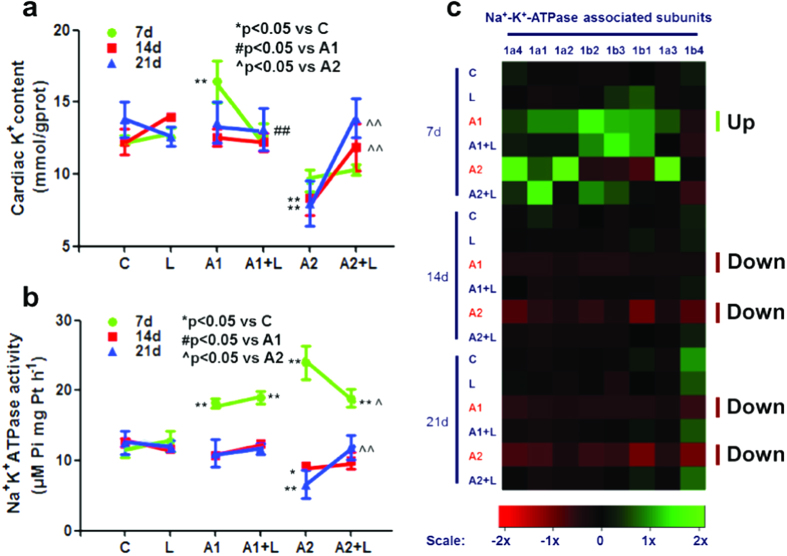
Effects of ATR and/or LYC on the modulation of K^+^ transfer channel. (**a**) The K^+^ content in cardiac myocytes; (**b**) The cardiac Na^+^-K^+^-ATPase activity; (**c**) The heat-map of 8 genes for Na^+^-K^+^-ATPase subunits. Values were expressed as mean ± S.D. Symbol for the significance of differences between the vehicle control and another: *P < 0.05, **P < 0.01. Symbol for the significance of differences between the 50 mg/kg ATR-treated group and 50 mg/kg ATR +5 mg/kg LYC treatment group: ^#^*P* < 0.05, ^##^*P* < 0.01. Symbol for the significance of differences between the 200 mg/kg ATR-treated group and 200 mg/kg ATR +5 mg/kg LYC treatment group: ^^^*P* < 0.05, ^^^^*P* < 0.01. The mRNA expression levels of Na^+^-K^+^-ATPase subunits are shown using the indicated pseudo color scale from −2x (green) to +2x (red) relative to values for mouse heart in the control group. The color scale represents the relative mRNA expression levels, with green indicating up-regulated genes, red indicating down-regulated genes, and black indicating unchanged genes.

The cardiac K^+^ content was significantly increased in the 50 mg/kg ATR-treated group at 7 days compared to the control group (Fig. 2a). However, there was a significant decrease in cardiac K^+^ content in the 200 mg/kg ATR-treated group at 7 days, 14 days and 21 days (Fig. 2a) compared to the control group. The cardiac K^+^ content was increased significantly in the LYC + ATR (200 mg/kg) group (*P* < 0.01) compared to the ATR (200 mg/kg) group at 14 days and 21 days. The serum Na^+^ content was significantly increased in the ATR-treated group at 21 days (*P* < 0.05). Additionally, the serum K^+^ content was significantly increased in the 200 mg/kg ATR-treated groups at 7 days, 14 days and 21 days (*P* < 0.01). Co-administration of LYC had no effect on ATR-induced changes in serum Na^+^ and K^+^ contents (see Supplementary Fig. S1a,b). The activi ties of cardiac Na^+^-K^+^-ATPase in the ATR- and/or LYC-treated groups at 7 days, 14 days and 21 days are shown in Fig. 2b. The Na^+^-K^+^-ATPase activity in the ATR-treated groups significantly increased (*P* < 0.01) at 7 days compared to the control group. Additionally, cardiac Na^+^-K^+^-ATPase activity was significantly decreased in the 200 mg/kg ATR-treated group compared to the control group at 21 days (*P* < 0.01). After mice were treated with LYC + ATR, a statistically significant improvement in the enzyme activities was observed (Fig. 2b). The maximal beneficial effect was observed for cardiac Na^+^-K^+^-ATPase activity after the administration of LYC + ATR (200 mg/kg) for 7 days, 14 days and 21 days (*P* < 0.01).

Unsupervised hierarchical clustering of the 8 Na^+^-K^+^-ATPase subunit mRNA expression levels showed a unique transcriptional response in the mice treated with ATR and/or LYC versus the control groups (Fig. 2c). Inspection of the resulting diagram identified clusters of ATR-regulated gene transcription in which 50 mg/kg and 200 mg/kg ATR appeared to have a similar, albeit more variable, effect. These results revealed a set of Na^+^-K^+^-ATPase subunits whose transcription was up-regulated to some extent by 50 mg/kg as well as 200 mg/kg ATR after 7 days of treatment (Fig. 2c), which was significantly different from the control group (see Supplementary Fig. S2a). Additionally, a set of Na^+^-K^+^-ATPase subunit genes was down-regulated following treatment with 50 mg/kg and 200 mg/kg ATR for 14 days and 21 days (Fig. 2c), which was significantly different from the control group (see Supplementary Fig. S2b,c). Co-administration of LYC had no effect on ATR-induced changes in Na^+^-K^+^-ATPase subunit transcript expression levels.

### Detection of Ca^2+^ content, Ca^2+^-ATPase activity and ATPase’s subunits transcription

**Figure 3:**
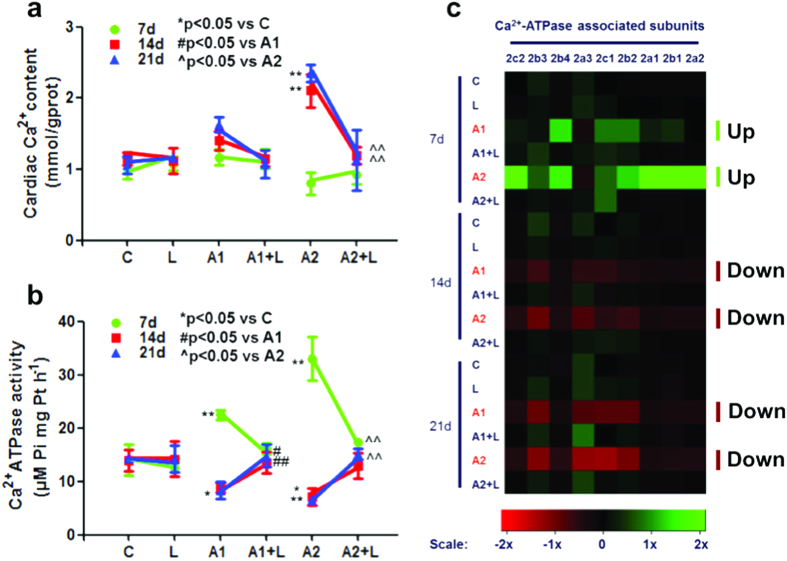
Effects of ATR and/or LYC on the modulation of Ca^2+^ transfer channel. (**a**) The Ca^2+^ content in cardiac myocytes; (**b**) The Ca^2+^-ATPase activity in cardiac myocytes; (**c**) The heat-map of 9 genes for Ca^2+^-ATPase subunits. Values were expressed as mean ± S.D. Symbol for the significance of differences between the vehicle control and another: *P < 0.05, **P < 0.01. Symbol for the significance of differences between the 50 mg/kg ATR-treated group and 50 mg/kg ATR +5 mg/kg LYC treatment group: ^#^*P* < 0.05, ^##^*P* < 0.01. Symbol for the significance of differences between the 200 mg/kg ATR-treated group and 200 mg/kg ATR +5 mg/kg LYC treatment group: ^^^*P* < 0.05, ^^^^*P* < 0.01. The mRNA expression levels of Ca^2+^-ATPase subunits are shown using the indicated pseudo color scale from −2x (green) to +2x (red) relative to values for mouse heart in the control group. The color scale represents the relative mRNA expression levels, with green indicating up-regulated genes, red indicating down-regulated genes, and black indicating unchanged genes.

The cardiac Ca^2+^ content was significantly increased in the 200 mg/kg ATR-treated mice after 14 days and 21 days of treatment (*P* < 0.01; Fig. 3a). After pre-treatment with LYC, the Ca^2+^ content returned to the normal level compared to the control group (Fig. 3a). In contrast, the Ca^2+^ content showed a marked decrease in the LYC + ATR (200 mg/kg) group at 14 days and 21 days compared to the 200 mg/kg ATR-treated group. There were no significant alterations in the serum Ca^2+^ level in the ATR and/or LYC treatment groups compared to the control group at 7 days, 14 days and 21 days (see Supplementary Fig. S1c). The alterations in the cardiac Ca^2+^-ATPase activity after ATR and/or LYC treatment are shown in Fig. 3b. A statistically significant increase in the Ca^2+^-ATPase activity was observed after 50 mg/kg and 200 mg/kg ATR exposure for 7 days (*P* < 0.01), whereas a significant decrease in the Ca^2+^-ATPase activity was observed after 50 mg/kg and 200 mg/kg ATR administration for 14 days (*P* < 0.05). Additionally, the activity of Ca^2+^-ATPase in the 200 mg/kg ATR-treated group was significantly decreased at 21 days (*P* < 0.01). However, administration of LYC along with ATR resulted in an increase in Ca^2+^-ATPase activity compared to the ATR-treated mice at 14 days and 21 days. A maximum protective effect was observed for the cardiac Ca^2+^ content and Ca^2+^-ATPase activity after administration of LYC+ATR (200 mg/kg) for 21 days (*P* < 0.01).

A heat map showing the levels of transcription of the 9 Ca^2+^- ATPase subunits in ATR- and/or LYC-treated mice is shown in Fig. 3c. Inspection of the resulting diagram identified clusters of ATR-regulated gene expression in the 50 mg/kg and 200 mg/kg ATR-treated groups that appeared to show a similar, albeit more variable, effect. The results revealed that the transcription of a set of Ca^2+^- ATPase subunits was up-regulated to some extent by 50 mg/kg as well as 200 mg/kg ATR treatment at 7 days (Fig. 3c), which was significantly different from the control group (see Supplementary Fig. S3a,b). Another set of genes was down-regulated by 50 mg/kg and 200 mg/kg ATR at 14 days and 21 days (Fig. 3c), which was significantly different from the control group (see Supplementary Fig. S3b,c). Co-administration of LYC had no effect on ATR-induced changes in Ca^2+^-ATPase subunit transcription.

### Detection of Mg^2+^ content and the activities of Mg^2+^-ATPase and Ca^2+^-Mg^2+^-ATPase

**Figure 4:**
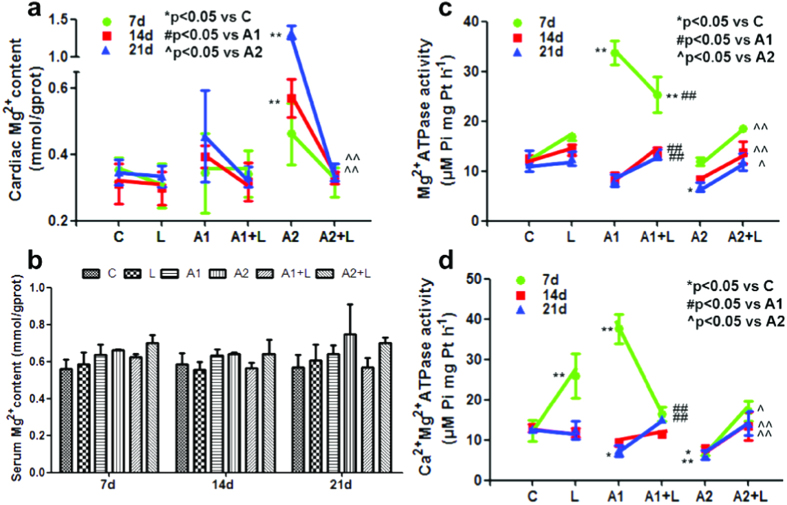
Effects of ATR and/or LYC on the modulation of Mg^2+^ transfer channel. (**a**) The Mg^2+^ content in cardiac myocytes; (**b**) The Mg^2+^ content in mouse serum; (**c**) The Mg^2+^-ATPase activity in cardiac myocytes; (**d**) The Ca^2+^-Mg^2+^-ATPase activity in cardiac myocytes. Values were expressed as mean ± S.D. Symbol for the significance of differences between the vehicle control and another: *P < 0.05, **P < 0.01. Symbol for the significance of differences between the 50 mg/kg ATR-treated group and 50 mg/kg ATR +5 mg/kg LYC treatment group: ^#^*P* < 0.05, ^##^*P* < 0.01. Symbol for the significance of differences between the 200 mg/kg ATR-treated group and 200 mg/kg ATR +5 mg/kg LYC treatment group: ^^^*P* < 0.05, ^^^^*P* < 0.01.

Cardiac Mg^2+^ content showed a significant increase in the 200 mg/kg ATR-treated group at 14 days and 21 days compared to the control group (Fig. 4a). After pre-treatment with LYC, the cardiac Mg^2+^ levels were significantly decreased at 14 days and 21 days compared to the ATR-treated group (*P* < 0.01; Fig. 4a). The serum Mg^2+^ content showed no significant alteration in the ATR-treated group compared to the control group (Fig. 4b).

Figure 4c shows a significant increase in Mg^2+^-ATPase activity in the 50 mg/kg ATR-treated group at 7 days compared to the control group (*P* < 0.01). A significant increase in Mg^2+^-ATPase activity in the 50 mg/kg ATR-treated group at 7 days (*P* < 0.05) and a significant decrease in Mg^2+^-ATPase activity in the 200 mg/kg ATR-treated group at 21 days (*P* < 0.05) were also observed. The cardiac Mg^2+^-ATPase activity was significantly increased in the LYC + ATR (200 mg/kg) treatment group compared to the 200 mg/kg ATR group at 7 days, 14 days and 21 days (*P* < 0.05). A maximum protective effect was observed in cardiac Mg^2+^-ATPase activity after administration of LYC + ATR (200 mg/kg) for 21 days (*P* < 0.001).

The results of Ca^2+^-Mg^2+^-ATPase activity after ATR treatment are presented in Fig. 4d. There was a significant increase on Ca^2+^-Mg^2+^-ATPase activity in the 50 mg/kg ATR-treated group at 7 days compared to the control group (*P* < 0.01). A maximum decrease in Ca^2+^-Mg^2+^-ATPase activity was observed in the ATR (200 mg/kg) group (*P* < 0.05) at 14 days and 21 days. The Ca^2+^-Mg^2+^-ATPase activity in the 50 mg/kg ATR group at 7 days was significantly altered in the LYC pre-treatment group (*P* < 0.05), which recovered to the normal level. A significant increase in the activity of this enzyme at 7 days, 14 days and 21 days was observed in the LYC + ATR (200 mg/kg) group compared to the ATR (200 mg/kg) group (*P* < 0.05). These results revealed that pre-treatment with LYC recovered the activities of Ca^2+^-Mg^2+^-ATPase and Mg^2+^-ATPase, which were altered by ATR exposure.

### PCA of cardiac ionic homeostatic modulation

**Figure 5:**
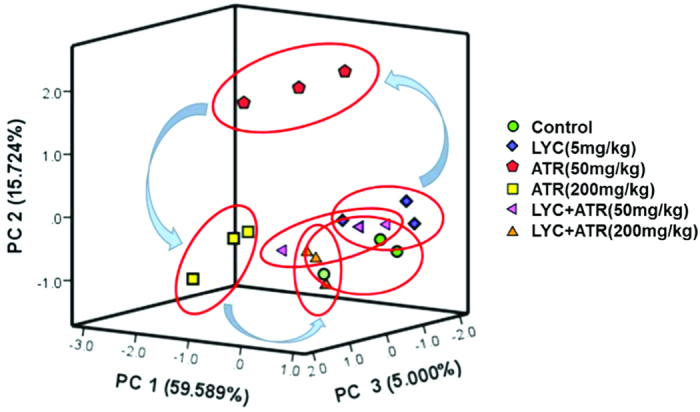
PCA of cardiac ionic homeostatic regulation after treated with ATR and/or LYC. PCA score plot results comparing biochemical parameters of 6 treatment groups.

Principal component analysis (PCA) for the relevant metabolites was performed with an analysis of variance (*P* < 0.05), and the results are depicted in Fig. 5. PC1, PC2 and PC3 conglomerated more than 80% of the total variance (see Supplementary Table 3S). Cross-validation allowed checking of the predictive power of the generated model using the Q2/R2 ratio as a measure of the reliability of the predictions. This ratio was above 0.8 for PC1, PC2 and PC3, suggesting that the generated model was consistent and valid (see Supplementary Table 3S).

To distinguish potential effects of biochemical parameters in mice receiving different doses of ATR, PCA was performed as an unsupervised pattern recognition method. The PCA scores showed that the dose-dependent separation within the ATR-treated animals was quite remarkable, and the changes in metabolic profiles from the controls to the low- and high-dose groups occurred in a counterclockwise direction; while clearly separated from the controls, there was some overlap among the control group, the LYC (5 mg/kg) group and the two ATR (50 and 200 mg/kg) with LYC (5 mg/kg) groups (Fig. 5). Taken together, these results showed that ATR exposure resulted in dose-dependent changes in biochemical parameters. The PCA scores showed that the time-dependent separation within the ATR-treated animals was quite remarkable, and the changes in metabolic profiles from 7 days to 14 days and 21 days occurred in a clockwise direction; however, these changes were clearly separated from the 7 day group (see Supplementary Fig. S5). Taken together, these results showed that ATR exposure resulted in time-dependent changes in biochemical parameters.

## Discussion

ATR is the most widely used pesticide in the world, and this contaminant can even be found in drinking water after treatment in the United States. People who drink water contaminated with ATR over the course of many years may experience problems with their cardiovascular system. In the present study, we investigated whether ATR exposure induced cardiac damage in mice. Our results showed that supplemental LYC alleviated the histopathologic changes as well as biochemical alterations mediated by ATR exposure. These results suggest that LYC protects the heart against ATR-induced cardiotoxicity. However, the chemopreventive potential of LYC against ATR exposure should be further elucidated in future studies.

Previous studies have indicated that ATR can be widely distributed and extensively metabolized in mice^18^. ATR exposure causes damage to multiple organs^6^, and our previous study showed that ATR could induce hepatic toxicity in mice^19^. The highest concentrations of ATR were recorded in the liver, which showed that the liver is one of the target organs involved in the metabolism of ATR. The bio-accumulation factors for ATR in the liver, muscle, heart, gonads and brain range from 0.9 to 20.0^5^. Indeed, ATR and/or its metabolites were found in heart tissues^5^, and our previous study showed that ATR induced significant ionic disorders in mouse livers^19^. The ionic content plays a role in a number of physiological processes, and proper ion concentrations are vital to ensure correct functioning of the entire body, especially the heart. Therefore, the liver and heart have been identified as the target organs for ATR-induced ionic disorders.

The bio-concentration of ATR was previously determined in the hearts of fish exposed to ATR^5^. In addition, ATR at concentrations between 10 mg/L and 20 mg/L caused retardations in organogenesis, a slowdown of movements, and functional disturbances of the heart and circulatory system in zebrafish^3^. Stasis of circulation, blood islands, titanic convulsions, a tube heart and mortality were observed in ATR-treated embryos with a concentration-response relationship^4^. The heart rate was also shown to be significantly decreased in snail embryos treated with ATR^20^. Jenny R. *et al.* found a significant dose-dependent increase in the percentage of ATR-exposed tadpoles with malformations of the circulatory system^21^. It was also reported that ATR promoted angiogenesis in the rat myocardium^22^. Significant hyperaemia and vesicular degeneration were observed in 50 mg/kg and 200 mg/kg ATR-exposed mice at 21 days in our study. The alterations in the pathological structure of cardiac myocytes and the concomitant alterations in CK levels indicated that ATR could induce cardiac myocyte dysfunction. ATR is metabolized and cleared rapidly in animals, while DACT is the major metabolite detected in urine, plasma, and tissues^17^. Our data suggested a significant dose-dependent and time-dependent effect on the ATR and DACT levels detected in mouse hearts.

LYC has attracted considerable attention because of its association with a decreased risk of certain chronic diseases, especial CVD and cancer^16^,^23^,^24^. Indeed, increased consumption of LYC is recommended for CVD prevention^25^. Recent studies have also indicated a role for tomato products in improving endothelial function and blood flow^26^. LYC is a hydrocarbon carotenoid that has recently received attention for its potential role in preventing CVD, and there is increasing evidence to suggest that LYC may lead to a reduction of intima-media thickness in vessel walls^27^. Considerable evidence has further suggested that LYC has significant antioxidant potential *in vitro* and may play a role in preventing CVD^12^,^28^. Indeed, intimal wall thickness and the risk of myocardial infarction were found to be reduced in subjects treated with LYC^9^. In another study, a tomato product inhibited lipaemia-induced postprandial oxidative and inflammatory responses^29^, which suggests that LYC may be important in preventing disease or that LYC is acting through some mechanism beyond that of autoxidation. In the past few years, we have gained greater knowledge of the mechanism and biological effects of LYC. However, while numerous effects of LYC have been shown, the molecular mechanism of action underlying the association between LYC and a reduced risk of CVD is not well understood. Indeed, the ability of LYC to reverse ATR-induced cardiac toxicity has not been studied in previous experiments. Consistent with our findings, LYC is thought to play a protective role in mice with dysregulated myocytes^12^. Moreover, our study demonstrated that LYC had a chemoprotective role against ATR-induced myocardial structural and functional damage in mice.

Because K^+^, Na^+^ and Ca^2+^ are involved in a number of physiological processes, proper ion concentrations are crucial to ensure correct functioning of the entire body, especially the heart. In the present study, we found that LYC did not interfere with normal ionic balance. LYC significantly regulated ATPase gene expression. Moreover, LYC ameliorated the ionic disorder that was induced by ATR. Fish previously exposed to ATR showed significant increases in Mg^2+^, Na^+^, and Ca^2+^, and it was concluded that under the conditions imposed in this study, ATR stimulated ionoregulatory growth and endocrine disturbance^30^. However, whether LYC can reverse myocardial ionic disorders induced by ATR remain unclear. In this study, ATR led to heart and serum ionic disorders, which induced structural alterations and dysfunction in cardiac myocytes. Furthermore, supplementation with LYC significantly modulated the ATR-induced changes in ionic levels in cardiac myocytes. These alterations in the pathological structure of cardiac myocytes indicated a role for ionic disorder in ATR-induced damage, and our results showed that ATR-induced ionic disturbances in mouse cardiac myocytes could be reversed by LYC treatment.

ATPases, which constitute a major category of ion transporters in the human body, have a variety of significant biological and pathological roles. These transporters are widely distributed throughout different organs of the body and have been best described in cardiac myocytes. ATPases play a fundamental role in regulating the transmembrane ionic balance to maintain proper function of the vasculature^31^, and many studies have shown that ionic disorders in the vasculature give rise to cardiac dysfunction. Recently, there has been greater focus on bench-to-bedside approaches, with genomic studies unveiling more promising therapeutic targets for novel therapies^32^. ATR exposure was found to significantly inhibit the activities of membrane ATPases such as Na^+^-K^+^-ATPase, Mg^2+^-ATPase, and Ca^2+^-ATPase in rats^33^. However, this effect showed a biphasic response, with an increase in the concentration or enzyme activity for a short time, followed by a decrease or inhibition of the enzymes after longer exposure to ATR. In addition, the time scale and the duration of the increased response and that of the decreased response depended on the concentration and toxicity of the pollutants in carp gills^34^. In our previous study, hepatic Na^+^-K^+^-ATPase activity was increased, whereas the activity of hepatic ATPases, such as Ca^2+^-ATPase, Ca^2+^-Mg^2+^-ATPase, and Mg^2+^-ATPase, was decreased^19^. In the present study, the activities of Na^+^-K^+^-ATPase, Ca^2+^-ATPase, Ca^2+^-Mg^2+^-ATPase, and Mg^2+^-ATPase were increased at 7 days for a short time in ATR-treated mice, whereas these levels were decreased at 14 days and 21 days after longer exposure to ATR. The inhibition of these enzymes showed a time-dependent and dose-dependent effect. These results indicate that ATR can induce ionic disorders by altering ATPases and contribute to cardiac dysfunction during intoxication. The current study further highlights the role of ATPases in various diseases. Research aimed at discovering novel therapeutic targets is an essential step forward for drug development and could potentially revolutionize pharmacological disease prevention and treatment. In the present study, LYC ameliorated the changes in ATPase activity that were induced by ATR, which suggests that LYC has a strong positive effect on cardiac myocyte ATPase function and ionic levels, thereby contributing to normal cardiac function.

**Figure 6:**
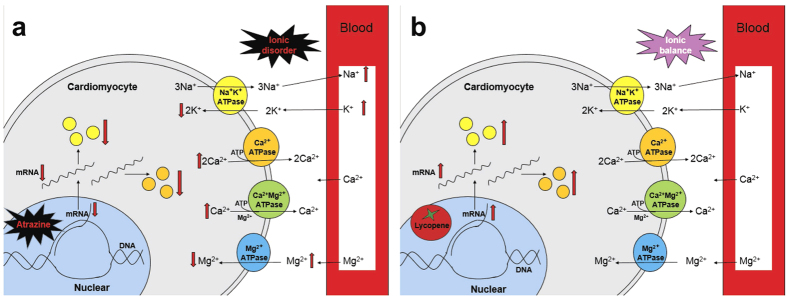
The pathway of ATR and LYC modulated ionic homeostasis in the cardiomyocytes. (**a**) ATR; (**b**) LYC.

Because K^+^, Na^+^ and Ca^2+^ are involved in a number of physiological processes, proper ion concentrations are crucial to ensure correct functioning of the entire body. The presence of K^+^ abnormalities worsens heart failure in patients, which increases risk for post-discharge morbidity or mortality^35^. In our previous study, hepatic K^+^ concentrations did not change significantly^19^, which indicated that the exchange of hepatic ions with serum ions reached equilibrium. Furthermore, cardiac K^+^ decreased significantly, while serum K^+^ increased significantly, which suggested that K^+^ was transported from the blood into the heart. In our study, ATR caused an increase in serum Na^+^ and K^+^ and a decrease in cardiac K^+^, which could be moderated by LYC. The mechanisms responsible for the balance between K^+^ intra- and extracellular concentrations play a crucial role in maintaining proper serum levels. Many ionic pumps and ion channels are regulated by hormones that are involved in Na^+^ and K^+^ homeostasis. The most important ionic pump is the Na^+^-K^+^-ATPase, which is responsible for the formation of the transmembrane potential and for maintaining the resting potential in all living cells; this pump actively transports K^+^ into and Na^+^ out of cells. Previous studies have revealed that the responses of Na^+^-K^+^-ATPase to ATR exposure occurred in the immune organs of common carp (*Cyprinus carpio* L.)^36^, the gills of fish^37^,^38^ and the kidneys of female minnows (*Gobiocypris rarus*)^39^. The effect of LYC on murine Na^+^ and K^+^ contents and cardiac Na^+^-K^+^-ATPase levels has not been reported in previous studies. In our study, mice exposed to ATR showed significant reductions in Na^+^-K^+^-ATPase activity, Na^+^-K^+^-ATPase-associated subunit transcription and cardiac K^+^ at all concentrations at 14 days and 21 days. This result could be explained by the fact that the isolated cardiac K^+^ content, Na^+^-K^+^-ATPase activity and subunit transcriptional levels in mice were already activated by LYC in our experimental conditions. Our results demonstrated that ATR delivered cardiotoxicity via the regulation of cardiac Na^+^-K^+^-ATPase activity and subunit transcription as well as disorders in K^+^ levels (Fig. 6a). However, LYC significantly protected the heart against ATR-induced cardiotoxicity. In particular, LYC modulated cardiac K^+^ homeostasis disturbance via regulation of Na^+^-K^+^-ATPase activity and subunit transcription (Fig. 6b).

One primary mechanism involved in ATR injury is the exacerbation of intracellular Ca^2+^, which causes damage to heart tissues. ATR exposure was accompanied by a significant decrease in ATPases and disturbed Ca^2+^ homeostasis in rat erythrocytes^40^. It has also been reported that ATR can induce Ca^2+^ transients in different cells^40^. It is well known that Ca^2+^ disorders cause dysregulated myocytes^41^, which is consistent with our findings. ATR can also induce Ca^2+^ release from intracellular compartments. Harmful effects of ATR on living organisms have resulted in the ability of ATR to induce intracellular Ca^2+^ release. Ca^2+^ levels in hepatic cells and cardiac cells were significantly increased, which suggested that Ca^2+^ was transferred from the blood into the liver and heart. However, the serum Ca^2+^ contents did not continue to change. Furthermore, the proteins associated with Ca^2+^ transfer include Ca^2+^-ATPase but also calbindins, the Na^+^/H^+^ pump and the calcium channel; thus, there may be other pathways that regulate Ca^2+^ and that require further investigation. In our study, LYC could maintain intracellular Ca^2+^ homeostasis in the heart. The mechanism of cardiac excitation-contraction coupling is based on the regulation of intracellular Ca^2+^ concentrations by Ca^2+^-ATPase in the sarcoplasmic reticulum of cardiomyocytes^42^,^43^,^44^. Substantial inhibition of this enzyme may lead to Ca^2+^ accumulation. The Ca^2+^-ATPase is the major active Ca^2+^ transport protein responsible for the maintenance of intracellular Ca^2+^ concentrations, and LYC was shown to reduce ATR-induced Ca^2+^ transients and decrease Ca^2+^-ATPase concentrations in murine hepatocytes in our previous study^19^. In this study, the role of ATR in the regulation of Ca^2+^ contents in mice hearts was also demonstrated. These observations may be explained by the decreased transcription and activity of Ca^2+^-ATPase in ATR-treated cardiac myocytes. Therefore, the up-regulation of Ca^2+^-ATPase activity may contribute to the maintenance of Ca^2+^ homeostasis by increasing Ca^2+^-ATPase-associated subunit mRNA levels (Fig. 6a). Moreover, LYC was demonstrated to regulate intracellular Ca^2+^ homeostasis by activating transcription of the Ca^2+^-ATPase- and Ca^2+^-ATPase-associated subunits in cardiac myocytes of mice (Fig. 6b). LYC up-regulated the Ca^2+^-ATPase activity and associated subunit expression levels to restore Ca^2+^ handling and consequently improve cardiac contractility/relaxation. Therefore, LYC has the potential to improve or prevent heart failure.

There is growing interest in the beneficial effect of Mg^2+^ on cardiovascular disorders. A number of cardiovascular disorders, including myocardial infarction, arrhythmias and congestive heart failure, have been associated with low extracellular or intracellular concentrations of Mg^45^,^46^. Mg^2+^ plays an essential role in a wide range of fundamental cellular reactions in patients with ischaemic heart disease, and Mg also controls cardiac excitability and maintains myocardial electrical stability in haemodialysis patients^47^. Experiments in animal models of myocardial infarction have provided evidence for early Mg overload during or after ischaemia^48^. Additionally, mitochondrial generation of ATP, which may be partly mediated by an increase in Ca^2+^ overload in the cytosol and/or mitochondria, can promote necrotic or apoptotic cell death in the ischaemia-reperfused heart^49^. The impairment of these transport systems in the cell may result in a continual increase in cytosolic Ca^2+^ levels, producing overactivation of cellular processes leading ultimately to cell death^50^. In our previous study, the hepatic Mg^2+^ concentration did not change significantly^19^, which indicated that the exchange of hepatic ions with serum ions reached equilibrium. In our current study, the cardiac Mg^2+^ levels were up-regulated significantly in ATR-treated groups. Thus, we suggest that because serum Mg^2+^ was unchanged, the increase in cardiac Mg^2+^ may be a response to the increase of Mg^2+^ outside the cardiomyocytes. In summary, we demonstrated that the alteration of Mg^2+^ induced by ATR was down-regulated by the Mg^2+^-ATPase (Fig. 6a). Moreover, LYC, which up-regulated the Mg^2+^-ATPase, maintained the Mg^2+^ balance. Because LYC improved cardiac function (Fig. 6b), our findings suggest that the Mg^2+^ balance has an important role in the heart-protective effect of LYC as a pre-conditioning agent.

In conclusion, ATR induced cardiotoxicity via the modulation of cardiac ATPase activity and changes in transcription of the pump subunits, leading to ionic disorder. However, supplementary LYC significantly prevented ATR-induced cardiotoxicity and alleviated the disturbance in cardiac ionic homeostasis via the regulation of ATPase activity and transcription of its subunits. Therefore, LYC showed significant chemopreventive potential against ATR-induced cardiotoxicity in mice. Nevertheless, further research is needed on the myocardial protective role of LYC and its ability to reduce morbidity and mortality in patients suffering from a variety of cardiac diseases.

## Materials and Methods

### Ethics statement

All experimental procedures were approved by the Institutional Animal Care and Use Committee of Northeast Agricultural University (NEAU). Methods were carried out in accordance with the approved guidelines of NEAU.

### Animals and experimental design

Male Kun-Ming mice (18–23 g) aged 21 postnatal days (PNDs) were used for the experimental study. Animals were housed under laboratory conditions at 22 ± 2 °C and were subjected to a controlled photoperiod (12 h light and 12 h dark) and relative humidity of 50 ± 15%. Pelleted food and water were provided *ad libitum*. The mice were acclimatized for 10 days before the start of the study. At PND 31, mice were assigned to the following experimental groups:

Group 1 (C), control animals treated with corn oil and distilled water for 21 days (n = 10); Group 2 (L), animals treated with 5 mg/kg LYC (North China Pharmaceutical Group, China) for 21 days (n = 10); Group 3 (A1), animals treated with 50 mg/kg ATR (Shandong Binnong Technology Co., Ltd. China) for 21 days (n = 10); Group 4 (A2), animals treated with 200 mg/kg ATR for 21 days (n = 10); Group 5 (A1 + L), animals treated with 50 mg/kg ATR and 5 mg/kg LYC for 21 days (n = 10); Group 6 (A2 + L), animals treated orally with 200 mg/kg ATR and 5 mg/kg LYC for 21 days (n = 10).

We chose this dose of ATR because doses higher than 50 mg/kg/day are considered toxic^51^. Previously, many studies have used 50 mg/kg/day and 200 mg/kg/day as toxic dosages^52^,^53^,^54^,^55^,^56^,^57^. Therefore, to study the effect of ATR on the hearts of mice, we treated animals by administering ATR at a dose of 50 mg/kg/day or 200 mg/kg/day for 21 consecutive days. Until now, no studies have examined the chemoprotective role of LYC against ATR-induced toxicity. A dose of 5 mg/kg LYC was chosen to alleviate various types of poison-induced toxicity in a large number of studies^58^,^59^,^60^,^61^. Thus, supplementation with LYC (5 mg/kg) served as the dosage to ameliorate ATR-induced toxicity. We also conducted a preliminary experiment before the formal experiments to demonstrate that the dosages of ATR and LYC were appropriate.

The entire experiment was repeated three times independently, and each group consisted of thirty animals with similar initial body weights. LYC was dissolved in corn oil for gavage administration for the treatment groups. ATR was dissolved in distilled water for gavage administration for the treatment groups. At the end of the experiment, the mice were weighed. Then, the mice were sacrificed by enucleation eye for blood, which was separated by centrifugation (2,400 g, 10 min) for serum samples. The heart tissue was excised immediately on an ice-cold plate and washed in physiological saline solution. Subsequently, the heart tissues were weighed immediately after the solution was removed with filter paper. The tissues and serum were stored at −80 °C for subsequent tests.

### Biochemical and histopathological analyses

Serum LDH and CK activities are frequently associated with cardiac toxic effects. Biochemical parameters, including LDH and CK, in the serum samples were measured using an automatic biochemistry analyser.

The hearts of the control and treated groups were fixed in 10% neutral buffered formalin and processed using routine histological techniques. After fixation with 10% buffered formalin, the heart was dehydrated, processed, and embedded in paraffin. Serial sections (5 μm) were prepared and stained with Harris’s haematoxylin and eosin (H&E) for histopathological evaluation by light microscopy (Nikon, Japan). The image acquisition parameters and microscope settings were kept the same throughout the process.

### Tissue ATR and DACT concentration assays

Mouse heart tissues were accurately weighed for 0.1 g and homogenized in aqueous acetic acid (0.01% v/v, 900 μL) as a 10% (w/v) mixture using a Fisher Scientific PowerGen Model 125 Homogenizer (Thermo Fisher Scientific, USA). Each tissue homogenate was vigorously vortexed for 3 min using a vortex oscillator (Thermo Fisher Scientific, USA), four volumes of ice-cold acetonitrile (typically 3200 μL) was added to the spiked homogenate (typically 800 μL), then was added to 0.4 g anhydrous sodium sulfate, mixed, and the samples placed on ice for 15 min. After centrifugation (10,000 rad/min, 8 min, 4 °C), the supernatants were transferred to clean test tubes and evaporated to dryness under nitrogen at room temperature. The residues were redissolved in 1:1 (v/v) 0.05% acetic acid/ethanol (500 μL), added 1 mL n-hexane to vortex. After centrifugation (8,000 rad/min, 6 min, 4 °C), the subnatant passed through a syringe filter (0.22 μm), and an aliquot (10 μL) was analyzed by LC/MS or LC/MSMS. Analyte recoveries from tissue extracts and method sensitivity were optimized by QuEchERS on the basis of Ross and Filipov’s method^18^,^62^. Recovery of analytes from tissues after this extraction procedure was typically ≥80%. DACT provided the poorest recovery (~80%) of all the tissue analytes examined, which was better than the previous study^62^. The concentrations of analytes in each tissue and/or biofluid are expressed in micrograms per kilogram (μg/kg).

LC/electrospray ionization/MS and LC/MSMS analysis of tissue extracts was performed on a Quattro Micro^TM^ API (Waters Corporation, USA). The mobile phase solvents were a blend of solvent A (0.1% v/v acetic acid in water) and solvent B (0.1% v/v acetic acid in methanol). After injection of the sample (10 μL) onto a Waters Acquity UPLC BEH C_18_ column (1.7 μm; 2.1 mm × 50 mm, Waters Corporation), the analytes were eluted with the following gradient program: 0 min (95% A, 5% B), 0.5 min (95% A, 5% B), 3 min (20% A), 3.5 min (20% A, 50% B), 5 min (95% A, 5% B). The flow rate was 0.25 mL/min. Ions were introduced into the MS by electrospray ionization in the positive ion mode. The MS single quadrupole was operated in select reaction monitoring mode with DACT and the triple quadrupole mass spectrometer was operated in multiple reaction monitoring mode throughout the chromatographic run. Calibration standards for each analyte were prepared in tissue matrices from naive untreated mice. The calibration standards in each matrix were worked up for LC/MS or LC/MSMS analysis in the exact manner as described for the unknown samples. Quality control samples were prepared with each sequence of samples to be run on the LC/MS or LC/MSMS. Blank samples (solvent blanks) were interspersed in the sequence to ensure no inters ample carryover was occurring.

The standard curves to calculate the concentration of ATR/DACT were made by the different concentration gradient of ATR standard (AccuStandard, USA, 100%) and DACT standard (AccuStandard, USA, 98.3%). The calibration curve of ATR was y = 227.414*x + 1.36837. And the calibration curve of DACT was y = 2459.73*x + 57071.8.

The detection and quantitation limits for ATR were evaluated using LC/MSMS. Additionally, the DACT in mouse tissues were detected by LC/MS. To estimate the limits of detection (LOD) and quantitation (LOQ) of each analyte in tissue extracts, the lowest calibration standard containing all of the target compounds was injected onto the LC/MS seven consecutive times to estimate the average base peak intensity for each analyte and to determine the standard deviation (S.D.) of the baseline noise over 100 consecutive scan events immediately adjacent to the analyte peak. The signal-to-noise (S/N) ratios in each analytical run were estimated using the following calculation: S/N = (base peak intensity of analyte = baseline noise intensity)/(S.D. of baseline noise intensity), and the average values were calculated. For each analyte, LOD and LOQ values were determined assuming S/N = 3 and S/N = 10, respectively.

### Ion concentrations in the heart and serum

Approximately one gram of heart tissue was minced and homogenized (ten times the weight of tissue) in physiological saline and then centrifuged (3,000 g for 10 min). The resulting clear supernatant was collected for ionic content and ATPase activity estimations. The cardiac K^+^, Ca^2+^ and Mg^2+^ concentrations and the serum Na^+^, K^+^, Ca^2+^ and Mg^2+^ concentrations were measured with detection kits (Nanjing Jiancheng Bioengineering Institute, China).

### Determination of protein content

Protein determinations were made using the dye-binding method of Bradford. Bovine serum albumin (BSA) was used to construct the standard curve.

### Cardiac ATPase activity assays

The activities of Na^+^-K^+^-ATPase, Ca^2+^-ATPase, Mg^2+^-ATPase and Ca^2+^-Mg^2+^-ATPase were determined using the appropriate assay kits (Nanjing Jiancheng Bioengineering Institute, China) according to the manufacturer’s instructions using 10% tissue homogenates. The activities of Na^+^-K^+^-ATPase, the Ca^2+^-ATPase and the Mg^2+^-ATPase were measured by quantifying the inorganic phosphorus (Pi) production from the conversion of ATP to ADP at 660 nm using the molybdenum blue spectrophotometric method and were expressed as μmolPi/mgprot/h. When one type of ATPase was tested, the inhibitors of other types of ATPase were added to depress the hydrolysis of phosphate radicals.

### Quantitative real-time polymerase chain reaction

RNA was extracted from cells using RANout reagent (Beijing Tiandi, Inc. China) according to the manufacturer’s instructions. For cDNA synthesis, 2.0 μg RNA was transcribed using the TransScript Reverse Transcriptase (Beijing TransGen Biotech Co. Ltd., China). Real-time quantitative PCR was performed using the GoTaq^®^ qPCR Master Mix (Promega, USA) according to the manufacturer’s protocol on BIOER LineGene 9620 (Hangzhou bioer Technology Co., Ltd. China). The primer sequences used for the gene expressions analyses are shown in Table S2. Lyceraldehyde-3-phosphate dehydrogenase (GAPDH) and β-actin was used as housekeeping controls to normalize the amounts of cDNA among the samples. Differences were calculated using the threshold cycle (Ct) and comparative Ct methods for relative quantification. The results were expressed as the relative expression of mRNA levels compared to control samples. The mRNA relative abundance was calculated according to the 2^−ΔΔCT ^63 method, and the results were normalized to the mean of β-actin and GAPDH.

### Statistical analysis

The results are expressed as the mean ± S.D. and were analysed with GraphPad Prism 5.0 (GraphPad Software, USA) and SPSS 19.0 software (SPSS Inc., USA). Statistical analyses were performed using one-way ANOVA followed by Tukey’s post hoc pairwise comparison. Asterisks (*) indicate statistically significant differences from the control group, *P < 0.05, **P < 0.01 and ***P < 0.001. Ranking of genes by degree of differential expression was analysed with a heat map using the R Programming Language version 3.2.1. In addition, PCA was used as an effective tool for simplifying the information from inter-correlated variables through linear transformation of the original variables into a few principal components. PCA was performed in this work to define the most important parameters, which could be used as key factors for individual variations using the same software. The observed relationships among the parameters were confirmed and quantified according to a Spearman’s test.

## Additional Information

**How to cite this article**: Lin, J. *et al.* The chemopreventive potential of lycopene against atrazine-induced cardiotoxicity: modulation of ionic homeostasis. *Sci. Rep.* **6**, 24855; doi: 10.1038/srep24855 (2016).

## Supplementary Material

Supplementary Figures
