# Supplementary material for: The chemopreventive potential of lycopene against atrazine-induced cardiotoxicity: modulation of ionic homeostasis
Source: Sci Rep. 2016 Apr 26;6:24855. doi: 10.1038/srep24855 (PMC4845055; doi:10.1038/srep24855)
Supplement: Supplementary Figures [file srep24855-s1.doc]

**The chemopreventive potential of lycopene against atrazine-induced cardiotoxicity: modulation of ionic homeostasis**

**Jia Lin^a^,***, **Hui-Xin Li ^b^,***, **Jun Xia^a^**, **Xue-Nan Li^a^**, **Xiu-Qing Jiang^a^, Shi-Yong Zhu^a^**, **Jing Ge^a^**, **Jin-Long Li^a^,****

^a^ College of Veterinary Medicine, Northeast Agricultural University, Harbin, 150030, People’s Republic of China

^b^ Division of Avian Infectious Diseases, State Key Laboratory of Veterinary Biotechnology, Harbin Veterinary Research Institute, Chinese Academy of Agricultural Sciences, Harbin, People’s Republic of China

*These authors contributed equally to this work.

**Corresponding author.

**Jin-Long Li**

Address: College of Veterinary Medicine, Northeast Agricultural University, Harbin, 150030, P. R. China

Tel: +86 451 55190407; E-mail address: Jinlongli@neau.edu.cn;


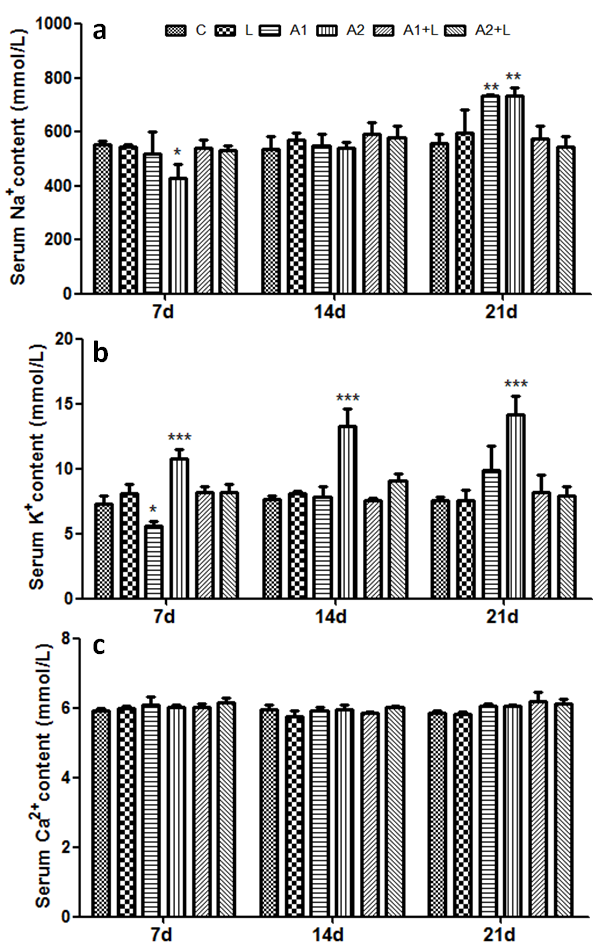


**Supplementary Figure S1. Effects of ATR and/or LYC on the Na^+^, K^+^, Ca^2+^ contents in mouse serum.**

(a) The Na^+^ content; (b) The K^+^ content; (c) The Ca^2+^ content. Values were expressed as mean ±S.D.. Symbol for the significance of differences between the vehicle control and another: * *P* < 0.05, ** *P* < 0.01.


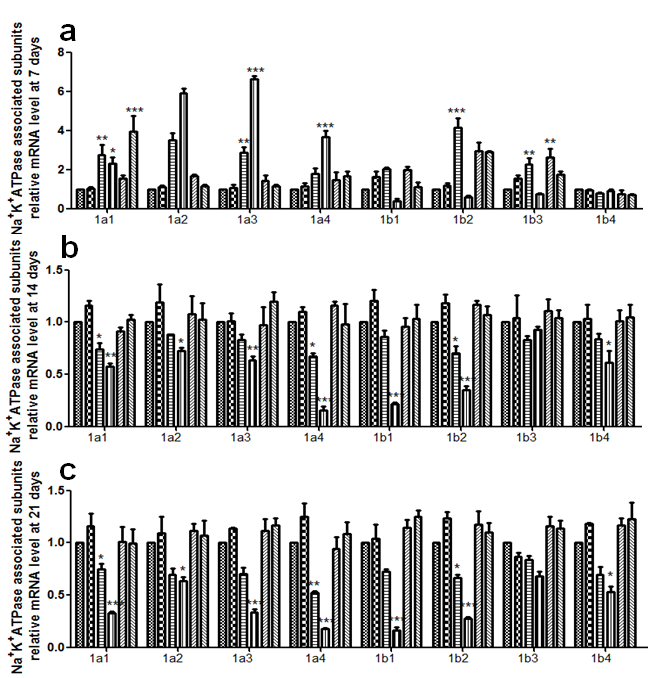


**Supplementary Figure S2.** **Effects of ATR and/or LYC on the mRNA expression levels of Na^+^-K^+^-ATPase subunits in mouse heart**.

(a) 7 days; (b) 14 days; (c) 21 days. Values were expressed as mean ± S.D.. Symbol for the significance of differences between the vehicle control and another: * *P* < 0.05, ** *P* < 0.01.


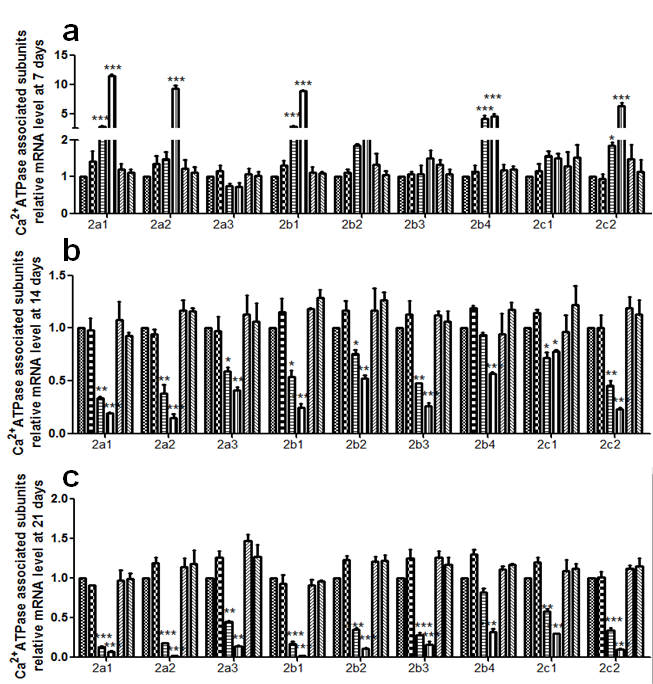


**Supplementary Figure S3.** **Effects of ATR and/or LYC on the transcription of a set of Ca^2+^- ATPase subunits in mouse heart**.

(a) 7 days; (b) 14 days; (c) 21 days. Values were expressed as mean ± S.D.. Symbol for the significance of differences between the vehicle control and another: * *P* < 0.05, ** *P* < 0.01.


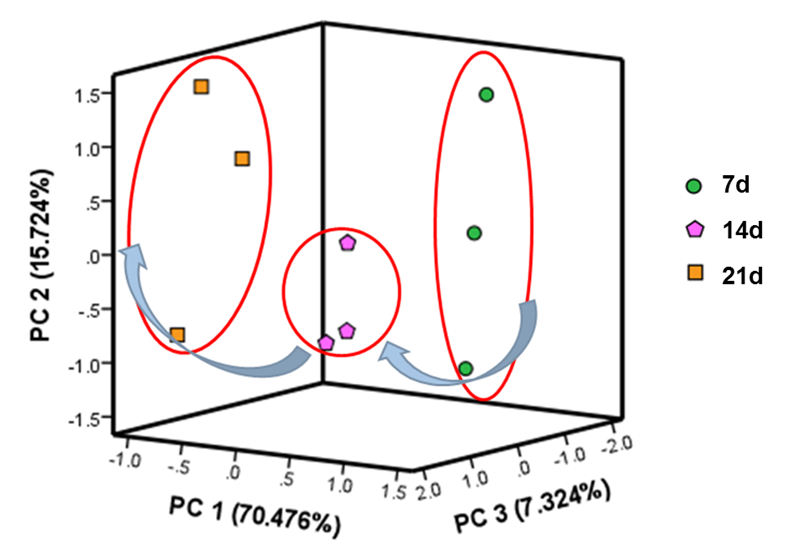


**Supplementary Figure S4.** **PCA score plot results comparing biochemical parameters at 3 time points**.

**Supplementary Table S1.** Summary weights of heart in mice at 21 days

| **Group** | **Body weight(g)** | **Heart weight (mg)** | **Relative weight (mg/g)** |
| --- | --- | --- | --- |
| **Control Group** | 33.07±2.782 | 182.5±23.4 | 5.53±0.741 |
| **LYC 5 mg/kg** | 34.88±1.107 | 190.3±25.9 | 5.45±0.728 |
| **ATR 50 mg/kg** | 33.70±2.761 | 202.1±31.5 | 6.00±0.829 |
| **ATR 200 mg/kg** | 31.48±1.288 | 194.4±18.7 | 6.17±0.578 |
| **LYC+ ATR 50 mg/kg** | 34.40±1.916 | 179.5±20.3 | 5.21±0.385 |
| **LYC+ ATR 200 mg/kg** | 34.49±2.713 | 183.0±44.0 | 5.50±0.826 |

Each value represents mean±SD (N=10, males).

**Supplementary Table S2.** **Oligonucleotides used for determination of ATPase subunits**.

|  | Gene | Forward Primer（5’→3’） | Reverse Primer（5’→3’） | Product length |
| --- | --- | --- | --- | --- |
| **housekeeper** | **ATCB** | CAAGAGAGGTATCCT GACCT | TGATCTGGGTCATCTTTTCAC | 188bp |
|  | **GAPDH** | CGTGCCGCCTGGAGAAACCTG | AGAGTGGGAGTTGCTGTTGAAGTCG | 140 bp |
| **Na^+^K^+^ ATPase associated subunits** | **1a1** | GGAGGCTTCTTCACTTACTT | GCACTACCACAATACTGACA | 199 bp |
|  | **1a2** | TTGGAGACGCGCAATATCTGT | CCATCACTGTCCGGTCACCT | 94 bp |
|  | **1a3** | GCCTTCTTTGTGAGTATCGTGGT | CCGTCTCCTCAAACAAGCCGAA | 127 bp |
|  | **1a4** | TCAACTACAAATAGACAGCCGAAA | AGGAGTTAGTACATTCGGTCCAT | 198 bp |
|  | **1b1** | AGCCCTGCATCATTATCAAGCTC | CCAAAGTACTCTATGTTCCCGACT | 178 bp |
|  | **1b2** | GGGCGATATTATGAGCAACCTGA | CTGCATAGAAGTTGATGACCCGAT | 178 bp |
|  | **1b3** | AGAACAGAAGAACCTCACAA | ATCAGTCACACCACTACATT | 118 bp |
|  | **1b4** | CTAGCCATCAGTCCTTACATGC | GCTGCCAAGTTTCAGGTTC | 124 bp |
| **Ca^2+^ ATPase associated subunits** | **2a1** | CCCAGACCAAGTTAAGCCACA | GAGAAGAATCCGCACAAGCAG | 124 bp |
|  | **2a2** | TGCTCAGATAAGACAGGCACAC | TGGCTTATCATCCTTTTGCACT | 158 bp |
|  | **2a3** | CCCACTGAAAGAAAGCATCTCGT | TTGTACACACCCAAGAAAGAGCTA | 163 bp |
|  | **2b1** | GAAGTCCATGAGCACAGTCCT | TGCCTCGCCATTAGCACT | 118 bp |
|  | **2b2** | TCATGATGCAGCTTTTCAACGA | ACGATCACTATCTGGATGGCGAAA | 133 bp |
|  | **2b3** | CCCAAAGCAGCCTAAGACCTTCC | ACACATTCCCACAGGCTTCACT | 149 bp |
|  | **2b4** | GAAGGTTCTGGACGGATG | CTGTTGAGTGGCTGGATT | 197 bp |
|  | **2c1** | GCCTGAGTATACTGGATCTGTTGT | CCCGGCTCCTTTCAACCT | 93 bp |
|  | **2c2** | CATCTGCTCTGACAAGACA | GTATCCGACACCACTGAC | 103 bp |

**Supplementary Table S3.** **Total Variance Explained of PCA.**

(a) Total Variance Explained of PCA for Figure 5; (b) Total Variance Explained of PCA for Figure S4.

Table S3a. Total Variance Explained of PCA for Figure 5.

| **Total Variance Explained** | | | | | | |
| --- | --- | --- | --- | --- | --- | --- |
| Component | Initial Eigenvalues | | | Extraction Sums of Squared Loadings | | |
|  | Total | % of Variance | Total | % of Variance | Total | % of Variance |
| 1 | 12.514 | 59.589 | 59.589 | 12.514 | 59.589 | 59.589 |
| 2 | 3.302 | 15.724 | 75.313 | 3.302 | 15.724 | 75.313 |
| 3 | 1.050 | 5.000 | 80.312 | 1.050 | 5.000 | 80.312 |
| 4 | .904 | 4.306 | 84.619 |  |  |  |
| 5 | .694 | 3.306 | 87.925 |  |  |  |
| 6 | .651 | 3.098 | 91.023 |  |  |  |
| 7 | .493 | 2.346 | 93.369 |  |  |  |
| 8 | .356 | 1.695 | 95.064 |  |  |  |
| 9 | .279 | 1.330 | 96.395 |  |  |  |
| 10 | .242 | 1.153 | 97.547 |  |  |  |
| 11 | .183 | .872 | 98.419 |  |  |  |
| 12 | .113 | .540 | 98.959 |  |  |  |
| 13 | .086 | .410 | 99.369 |  |  |  |
| 14 | .070 | .334 | 99.703 |  |  |  |
| 15 | .034 | .164 | 99.867 |  |  |  |
| 16 | .020 | .097 | 99.964 |  |  |  |
| 17 | .008 | .036 | 100.000 |  |  |  |
| 18 | 3.305E-16 | 1.574E-15 | 100.000 |  |  |  |
| 19 | 8.973E-17 | 4.273E-16 | 100.000 |  |  |  |
| 20 | 5.591E-17 | 2.662E-16 | 100.000 |  |  |  |
| 21 | -1.798E-16 | -8.564E-16 | 100.000 |  |  |  |
| Extraction Method: Principal Component Analysis (PCA). | | | | | | |

Table S3b. Total Variance Explained of PCA for Supplementary Figure S4.

| **Total Variance Explained** | | | | | | |
| --- | --- | --- | --- | --- | --- | --- |
| Component | Initial Eigenvalues | | | Extraction Sums of Squared Loadings | | |
|  | Total | % of Variance | Total | % of Variance | Total | % of Variance |
| 1 | 9.867 | 70.476 | 70.476 | 9.867 | 70.476 | 70.476 |
| 2 | 1.854 | 13.244 | 83.720 | 1.854 | 13.244 | 83.720 |
| 3 | 1.025 | 7.324 | 91.044 | 1.025 | 7.324 | 91.044 |
| 4 | .549 | 3.920 | 94.963 |  |  |  |
| 5 | .462 | 3.297 | 98.260 |  |  |  |
| 6 | .138 | .988 | 99.248 |  |  |  |
| 7 | .073 | .519 | 99.767 |  |  |  |
| 8 | .033 | .233 | 100.000 |  |  |  |
| 9 | 5.832E-16 | 4.166E-15 | 100.000 |  |  |  |
| 10 | 2.355E-16 | 1.682E-15 | 100.000 |  |  |  |
| 11 | 1.189E-16 | 8.492E-16 | 100.000 |  |  |  |
| 12 | -2.021E-17 | -1.444E-16 | 100.000 |  |  |  |
| 13 | -2.176E-16 | -1.554E-15 | 100.000 |  |  |  |
| 14 | -3.218E-16 | -2.298E-15 | 100.000 |  |  |  |
| Extraction Method: Principal Component Analysis (PCA). | | | | | | |
